# Tissue and circulating microRNAs as biomarkers of response to obesity treatment strategies

**DOI:** 10.1007/s40618-020-01453-9

**Published:** 2020-10-28

**Authors:** G. Catanzaro, T. Filardi, C. Sabato, A. Vacca, S. Migliaccio, S. Morano, E. Ferretti

**Affiliations:** 1 Department of Experimental Medicine, Policlinico Umberto I, “Sapienza” University of Rome, Viale del Policlinico 155, 00161 Rome, Italy; 2 Department of Movement, Human and Health Sciences, “Foro Italico” University of Rome, Rome, Italy

**Keywords:** Obesity, microRNA, Weight loss, Diet, Bariatric surgery, Obesity treatment

## Abstract

**Background:**

Obesity, characterized by an increased amount of adipose tissue, is a metabolic chronic alteration which has reached pandemic proportion. Lifestyle changes are the first line therapy for obesity and a large variety of dietary approaches have demonstrated efficacy in promoting weight loss and improving obesity-related metabolic alterations. Besides diet and physical activity, bariatric surgery might be an effective therapeutic strategy for morbid obese patients. Response to weight-loss interventions is characterised by high inter-individual variability, which might involve epigenetic factors. microRNAs have critical roles in metabolic processes and their dysregulated expression has been reported in obesity.

**Aim:**

The aim of this review is to provide a comprehensive overview of current studies evaluating changes in microRNA expression in obese patients undergoing lifestyle interventions or bariatric surgery.

**Results:**

A considerable number of studies have reported a differential expression of circulating microRNAs before and after various dietary and bariatric surgery approaches, identifying several candidate biomarkers of response to weight loss. Significant changes in microRNA expression have been observed at a tissue level as well, with entirely different patterns between visceral and subcutaneous adipose tissue. Interestingly, relevant differences in microRNA expression have emerged between responders and non-responders to dietary or surgical interventions. A wide variety of dysregulated microRNA target pathways have also been identified, helping to understand the pathophysiological mechanisms underlying obesity and obesity-related metabolic diseases.

**Conclusions:**

Although further research is needed to draw firm conclusions, there is increasing evidence about microRNAs as potential biomarkers for weight loss and response to intervention strategies in obesity.

## The challenges of obesity treatment

Obesity is a common condition that has been consistently linked to an increased risk of developing a wide range of disorders, such as metabolic syndrome (MS), type 2 diabetes (T2D), cardiovascular diseases (CVD), non-alcoholic fatty liver disease (NAFLD), musculoskeletal diseases and some cancers [1–4]. Over the last decades, the prevalence of this condition has dramatically risen, reaching pandemic proportions. Globally, it has been estimated that nearly 39% of adults are overweight and 13% are obese [5]. Obesity is defined as excessive fat accumulation ensuing from a chronic imbalance between calorie intake and energy expenditure, which is linked to unhealthy diet habits and physical inactivity [6]. Excess energy is stored as triglycerides in white adipose tissue (WAT), which comprises subcutaneous adipose tissue (SAT) and visceral adipose tissue (VAT). Although adipocytes display similar functions in SAT and VAT, the latter is more markedly associated with abnormal metabolic profiles, directly contributing to insulin resistance [7, 8]. Specifically, in vitro studies have reported a more consistent release of free fatty acids (FFA) in VAT due to a higher lipolytic activity, which is able to induce insulin resistance impairing insulin signalling [9, 10]. It has been observed that VAT expresses several pro-inflammatory and anti-inflammatory mediators which are critically involved in the maintenance of immune metabolic homeostasis [11]. Furthermore, the greater number of macrophages in VAT compared to SAT leads to a more marked production of pro-inflammatory cytokines, significantly contributing to the chronic low grade inflammation typical of obesity and metabolic diseases [12, 13].

Despite the advances in therapeutic strategies, obesity management remains highly challenging. Lifestyle modification is the primary approach for weight loss and fat mass reduction. Indeed, dietary energy restriction and increased energy expenditure through physical activity are the well-known cornerstones of obesity treatment [14]. Specifically, energy restriction is crucial for the weight-loss phase, while physical activity is essential for long-term maintenance. A decrease of at least 5% of the initial body weight is considered clinically significant and can be effectively achieved by lowering the daily caloric intake by 500 kcal [15]. Of note, exercise without dietary restrictions is able to achieve only 3–5% weight loss in patients with obesity [16]. A wide variety of dietary intervention patterns, such as Mediterranean diet, low carbohydrate diet, low-fat diet and ketogenic diet (KD), which significantly differ in the proportions of macronutrients, have been extensively evaluated in clinical trials and displayed great efficacy in reducing body weight and in improving metabolic abnormalities in obese patients [17–19]. On the other hand, bariatric surgery is a highly effective strategy for weight loss and comorbidities improvement in morbidly obese patients when life style intervention fails. Several surgical procedures based on restrictive or malabsorptive approaches are currently available. Specifically, laparoscopic sleeve gastrectomy (LSG) and laparoscopic adjustable gastric banding (LAGB) significantly restrict food intake, while Roux-en-Y gastric by-pass (RYGB) is mainly malabsorptive [20]. The most frequent complications of bariatric surgery include dumping, nausea, vomiting, diarrhoea, infections, stenosis, bleeding, increased risk of alcohol abuse after surgery, and perioperative death.[21, 22].

Despite the different therapeutic options, response to weight-loss programmes is hallmarked by high inter-individual variability, which might be partly explained by epigenetic factors [23–25]. In light of this, the development of novel tools based on nutrigenetic and nutrigenomic information is of utmost importance in the development of customized approaches in obesity management [26]. Thus, great efforts are being made to identify valuable predictors of response to diet or bariatric surgery interventions. Recently microRNAs, a class of small non-coding RNAs that regulate gene expression, have gained much attention not only as regulators of biological processes but also as prognostic biomarkers in obesity management.

## General aspects of microRNAs

microRNAs are small (19–25 nucleotides) non-coding single stranded RNAs that function destabilizing or depleting target mRNAs [27]. Since their discovery in *Caenorhabditis elegans* in 1993 they have been described in many other species, even viruses, and to date 2654 human mature microRNA sequences have been identified (miRbase version 22.1 released in October 2018) [28–31]. Their dysregulation has been described in the context of many pathological processes, spanning from cancer to neurological and metabolic diseases. Therefore, although their function has not been yet completely defined, their huge number and wide species distribution suggest a crucial role in gene regulation [32, 33].

microRNAs biogenesis, secretion and function involve many complex molecular events, not completely understood yet. microRNA genes are usually transcribed by RNA polymerase II and sometimes by RNA polymerase III. The pri-miRNA sequence is capped at the 5′ and polyadenylated at the 3′ end and recognized by the Microprocessor complex, constituted by the *Di George Syndrome Critical Region 8* (DGCR8) nuclear protein and the RNAse III Drosha. This first nuclear maturation step releases a precursor microRNA, called pre-miRNA. The protein Exportin-5 is then responsible for the translocation from the nucleus to the cytoplasm, where the pre-miRNA is cleaved by the RNAse III Dicer with the production of a double strand microRNA of about 22 nucleotides in length. After loading onto Ago2, a member of the Argonaute (Ago) protein family, the microRNA is included into the RNA-induced silencing complex (RISC), a big ribonucleoprotein effector complex. The binding with Ago2 favours the most stable strand, while the passenger strand is degraded to produce a mature RISC. The main mechanisms responsible for the target mRNA silencing are mRNA degradation and translational repression. Specifically, if the sequence homology between the microRNA and its target mRNA is complete, Ago proteins degrade the target mRNA. Otherwise, if the sequence homology is only partial, there is only translational repression [34, 35].

Although the cellular compartment is the site of microRNA production and action, recent evidence demonstrated that microRNAs can also act into the extracellular compartment after secretion. One of the most intriguing hypothesis related to their function and origin sustains that extracellular microRNAs are involved in cell-to-cell communication. Circulating microRNAs have been detected in different biological fluids, such as serum, plasma and urine [36, 37]. To date, it has been demonstrated that microRNAs can be packaged into shedding vesicles and exosomes or coupled with high-density lipoproteins (HDL) and low-density lipoproteins (LDL) or Ago proteins, and actively secreted by cells. In addition, microRNAs can be passively secreted after incorporation in apoptotic bodies [38].

In addition, microRNA encapsulation into extracellular vesicles is a well-structured and defined process. The microvesicle-packed microRNAs do not simply mirror the cell of origin repertoire, since some microRNAs are preferentially exported or retained in cells [39].

Importantly, thanks to the circulating microRNA association with both protein complexes and/or to their packing into extracellular vesicles, microRNAs show high stability into the extracellular environment, rendering not only very stable extracellular molecules, but also capable of acting as diagnostic, prognostic and therapeutic biomarkers [40–42].

Moreover, microRNAs play key roles in physiological and pathological processes. They are important in fat cell formation and in the regulation of metabolic and endocrine functions. Indeed, the integral role of microRNAs in adipose tissue is emerging from studies demonstrating that the inhibition of Drosha and Dicer in human mesenchymal stem cells (MSC) inhibited the differentiation into adipocytes, conversely Dicer in 3T3-L1 cells inhibited adipogenesis [43, 44]. Other studies showed that microRNA are involved in the regulation of adipogenesis, acting as stimulators (i.e. miR-21, miR-26b, miR-30, miR-103, miR-143, miR-148, miR-181a, miR-199a, miR-378) or inhibitors (i.e. let7, miR-22, miR-125a, miR-224) of human adipocyte differentiation programmes [45–51]. As regards human obesity, several studies identified dysregulated microRNAs. In particular, many microRNAs were differentially regulated in WAT of obese subjects compared to lean human subjects [52–54]. Finally, several microRNAs are known to influence lipolytic activity [55–57] and adipokine production in fat cells [58, 59].

## microRNAs and weight loss: evidence from lifestyle intervention studies

Interestingly, compelling evidence suggests that differences in the outcomes of dietary intervention might be related to epigenetic factors [25]. The role of microRNAs as biomarkers of weight loss after dietary and lifestyle interventions has been extensively investigated in a wide number of studies, exploring microRNA expression at a systemic level (white blood cells, serum and plasma) and in adipose tissue (Table 1).

**Table 1:** Studies that evaluated microRNA expression change after lifestyle intervention strategies

Study	Population	Intervention	Source		Regulated miRNAs	Role/target
Milagro et al. 2013 [60]	10 obese women	8-week hypocaloric diet	PBMC		miR-935, miR-4772 (↑) miR-376b (↓)	Biomarkers of weight loss
Marques-Rocha et al. 2016 [61]	40 MS	8-week hypocaloric Mediterranean diet	WBC		miR-155-3p (↓) let-7b (↑)	Biomarkers of improvement in quality of diet and weight loss (155-3p)
Garcia-Lacarte et al. 2018 [62]	96 MS	8-week hypocaloric diet	WBC		miR-612, miR-1976 (↑)	Biomarkers of high response to diet/TP53 and CD40
Garcia-Lacarte et al. 2019 [63]	96 MS	12-week hypocaloric diet	WBC		miR-548q, miR-1185-1 (↑)	Biomarkers of high response to diet/GSK3B
Hess et al. 2020 [65]	85 obese/overweight	12-week hypocaloric diet	Serum		miR-222-3p (↑) miR-122-5p, miR-193a-5p (↓)	Biomarkers of weight loss and MS/glucose metabolism
Giardina et al. 2019 [71]	103 obese	6-month LGI (*n* = 36), HGI (*n* = 36), LF (*n* = 31) diet;	Plasma		(↓) in LGI vs HGI diet: miR-139-3p, miR-411, miR-432, miR-99b, miR-340, miR-423-5p, miR-361, let-7c (↓) in LF diet: miR-139-3p	Association with specific dietary patterns/Lipid metabolism
Assman et al. 2020 [72]	78 obese 25 CTRL	high-protein diet (*n* = 38); LF diet (*n* = 40)	Plasma		LF diet: miR-130a-3p, miR-142-5p, miR-144-5p, miR-15a-5p, miR-221-3p, miR-29c-3p (↓) miR-22-3p (↑)	Biomarkers of response to LF diet/PPAR-γ, PPAR- α, SIRT1 (miR-22-3p)
Manning et al. 2019 [75]	80 obese women 80 CTRL women	4-week very-low-calorie diet	Plasma		miR-126, miR-375, miR-376, miR-499, miR-642 (↑) miR-208, miR-433 (↓)	Biomarkers of weight loss/glucose metabolism, inflammation, angiogenesis, cell death
Cannataro et al. 2019 [76]	36 obese	6-week KD	Plasma		Women: miR-148b-3p, miR-26a-5p, miR-520h, miR-548d-3p Both sexes: miR-30e-5p, miR-502-5p, miR-590-5p, miR-644a, let-7b-5p, miR-143-3p, miR-504-5p	Sex differences in microRNA expression after KD/ inflammation, immunity, glucose metabolism
Margolis et al. 2017 [77]	16 elderly men	28-day hypocaloric diet	Serum		miR-133a-3p, miR-133b (↑)	Biomarkers of reduced skeletal-muscle regeneration
Parr et al. 2016 [78]	111 obese	16-week diet (HPHC/HPMC/CON) and physical activity	Plasma		miR-140, miR-221, miR-223 (↑) low responders: miR-935 (↑)	Biomarkers of response to intervention
Donghui et al. 2019 [79]	37 obese male adolescents 10 CTRL	6-week hypocaloric diet and aerobic exercise	Serum	miR-126 (↑)		Biomarker of weight loss/endothelial function
Kristensen et al. 2017 [80]	38 obese	15-week hypocaloric diet and physical activity	SAT		miR-29a-3p, miR-29a-5p (↑) miR-20b-5p, miR-454-3p (↓)	Glucose uptake, lipid metabolism, energy homeostasis
Giardina et al. 2018 [84]	8 obese	6-month LGI (*n* = 3), HGI (*n* = 3), LF (*n* = 2) diet;	SAT		LGI diet: miR-551b, miR-221, miR-378, let7a (↓) HGI diet: miR-1276, miR-132, miR-29a (↓) LF diet: miR-661, miR-1179, miR-132, miR-221, miR-29a, miR-378 (↓)	Association with metabolic and body composition parameters

MS, metabolic syndrome; PBMC, peripheral blood mononuclear cells; WBC, white blood cells; GSK3B, glycogen synthase kinase-3 B; LGI, low glycaemic index; HGI, high glycaemic index; LF, low fat; CTRL, control subjects; PPAR-γ, peroxisome proliferator-activated receptor γ; PPAR-α, peroxisome proliferator-activated receptor α; SIRT1, sirtuin-1; KD, ketogenic diet; HPH, high dairy protein and carbohydrate; HPMC, high dairy protein and moderate carbohydrate; CON, low dairy protein and high carbohydrate; SAT, subcutaneous adipose tissue

### microRNA expression in white blood cells

In 2013, Milagro et al., characterized microRNA expression in peripheral blood mononuclear cells (PBMC) from 10 obese women undergoing an 8-week energy-restricted diet. The population was further categorized as responder (R, > 5% body mass loss, *n* = 5) and non-responder (NR, < 5% body mass loss, *n* = 5) to the intervention. At baseline, the study revealed a different expression of five microRNAs between the two groups. Specifically, in the NR group at baseline, miR-935 and miR-4772 were up-regulated, whereas miR-223, miR-224 and miR-376b were down-regulated. Notably, miR-935, miR-4772 and miR-376b also showed a relevant association with the magnitude of weight loss, being therefore valuable candidate biomarkers for weight loss and response to diet [60]. Subsequently, Marques-Rocha et al., focused on the modulation of the expression of nine selected inflammation-related microRNAs in white blood cells of 40 patients affected by MS after an 8-week energy-restricted Mediterranean diet. Two microRNAs showed a statistically significant change after the dietary intervention; specifically, miR-155-3p strongly decreased, while let-7b was significantly up-regulated. In addition, the authors showed that let-7b, miR-125b, miR-130a, miR-132-3p and miR-422b were positively associated with the Health Eating Index (HEI) improvement, a scoring metric of diet quality, and miR-155-3p also with weight loss [61]. Another study evaluated microRNA changes in white blood cells obtained from patients with MS enrolled in the RESMENA (Metabolic Syndrome Reduction in Navarra) nutritional trial, after two different energy-restricted dietary interventions. The enrolled subjects were classified as high responders (HR) and low responders (LR), when weight loss after 8 weeks was respectively higher or lower than 8%. Microarray analyses were performed to detect microRNA methylation (31 LR vs 16 h) and expression (14 LR vs 10 h) before and after intervention. Six microRNAs (miR-1237, miR-1976, miR-642, miR-636, miR-612, miR-193B) were identified as both hypomethylated and over-expressed in HR. Notably, miR-612 and miR-1976 were the most hypomethylated and over-expressed, respectively. The bioinformatics analysis revealed TP53 and CD40 as miR-612 and miR-1976 targets, respectively. Both of them were modulated after the dietary intervention, comparing HR to LR, suggesting a role of these microRNA/gene axes in MS and obesity [62]. The same authors further reported an up-regulation of miR-548q and miR-1185-1 in HR, compared to sex and gender-matched LR. In functional assay, miR-548q and miR-1185-1 reduced glycogen synthase kinase-3 B (GSK3B) gene expression, targeting its mRNA [63]. GSK3B is known to be involved in pro-inflammatory responses by promoting the expression of pro-inflammatory cytokines such as IL-1ß, IL-6 or TNF-α [64]. Accordingly, the authors reported a negative correlation between miR-1185-1 expression and serum levels of IL-6 [63].

### Circulating microRNAs

Several studies focused on the modulation of blood circulating microRNA levels after dietary interventions, comparing the effects of different dietary plans, with variable proportions of macronutrients.

Hess et al., evaluated the expression of selected serum microRNAs associated with obesity and MS in obese and overweight patients (*n* = 85), before and after a 12-week hypocaloric diet. The dietary intervention consisted of a 500 kcal/day restriction and a baseline randomization to fibre supplements or placebo. Circulating levels of miR-222-3p significantly increased after weight loss, while miR-122-5p and miR-193a-5p were reduced. At baseline, the concentrations of miR-122-5p and miR-193a-5p were higher in the presence of MS and positively correlated with markers of insulin resistance and body composition, such as HOMA-IR (homeostasis model assessment of insulin resistance), waist circumference and visceral fat mass [65]. Notably, the up-regulation of circulating miR-122-5p has been linked to NAFLD and its reduction after weight loss could therefore reflect an improvement in liver health [66]. As miR-122-5p and miR-193a-5p correlated with insulin resistance parameters, their reduction after intervention might mirror an improvement in glucose metabolism as well. MiR-222-3p has been previously linked to T2D and gestational diabetes (GDM), although its role in regulating glucose metabolism has yet to be elucidated [67–69]. Overall, these results suggest that specific microRNA patterns of expression might hallmark metabolically unhealthy obesity.

Ortega et al., investigated selected plasma circulating microRNAs in nine obese patients following a 14-week energy-restricted diet. However, the study revealed no significant associations between weight loss (17% reduction in body mass) and circulating microRNAs (miR-520c-3p, miR-15a, miR-590-5p, miR-126, miR-636, miR-625) [70].

Giardina et al., analysed plasma microRNA expression before and 6 months after three energy-restricted diets. Specifically, a discovery cohort of eight patients that underwent high-throughput screening was sub-grouped into three categories: moderate carbohydrate and low glycaemic index diet (LGI, *n* = 3), moderate carbohydrate and high glycaemic index diet (HGI, *n* = 3) and low fat and high glycaemic index diet (LF, *n* = 2). The authors identified eight differentially expressed microRNAs (miR-139-3p, miR-411, miR-432, miR-99b, miR-340, miR-423-5p, miR-361 and let-7c), whose significantly dysregulated levels were confirmed in a wider cohort of 103 patients (LGI, *n* = 36; HGI, *n* = 36; LF, *n* = 31). All these microRNAs were down-regulated in the LGI compared to the HGI group, with miR-361 showing the greatest reduction. Besides this, miR-139-3p was down-regulated both in the HGI and in the LF group compared to the baseline and positively correlated with both LDL-cholesterol and total cholesterol. MiR-340 was down-regulated only in the HGI group and positively correlated with triglycerides changes. MiR-432 and miR-423 were down-regulated only in the LF group and the latter had the same correlations reported for miR-139-3p. Finally, let-7c correlated with all lipid parameters [71].

The expression of 86 circulating obesity-related microRNAs has been explored in obese patients, randomly assigned to a moderately high-protein diet (*n* = 38) or a low-fat diet (*n* = 40), and in normal weight controls (*n* = 25). Among the differentially expressed microRNAs, in obese subjects compared to controls seven microRNAs (miR-130a-3p, miR-142-5p, miR-144-5p, miR-15a-5p, miR-22-3p, miR-221-3p and miR-29c-3p) were significantly associated with response to low-fat diet and strongly discriminated between responders and non-responders. In particular, a correlation between miR-22-3p expression and the entity of weight loss has been observed [72]. Interestingly, several regulators of fatty acid metabolism, such as peroxisome proliferator-activated receptor α (PPAR-α) and sirtuin-1 (SIRT1), are confirmed targets of miR-22 [73, 74].

A global microRNA profiling has been performed in plasma samples obtained before and after weight loss from obese women undergoing a 4-week very-low-calorie diet and from age-matched lean women. The expression of ~ 800 microRNAs has been evaluated (*n* = 8 samples each group) and validated in a cohort of 80 samples for each group. 21 microRNAs were found to be significantly dysregulated in obese women at baseline compared to lean women, their predicted targets belonged to pathways involved in glucose metabolism, inflammation, angiogenesis and cell death. Interestingly, the expression of seven microRNAs (miR-126, miR-208, miR-375, miR-376, miR-433, miR-499, miR-642) significantly changed in obese women after weight loss becoming similar to lean women, suggesting that an acute weight loss programme might effectively reverse the marked dysregulation of circulating microRNAs in obese subjects [75].

The effects of a KD on biochemical parameters, body composition and plasma microRNA profile has been evaluated in 36 obese subjects after 6 weeks of intervention. The KD is a very low carbohydrate nutritional regimen, characterized by less than 30–50 g of carbohydrates per day. In this study a biphasic KD was carried out: in the first 3-week phase, carbohydrate content was lower than 30 g per day, while in the last three weeks it reached 120 g per day. During the subsequent 6-weeks of follow-up, serum samples were collected for a high-throughput screening analysis on 799 microRNAs. The dietary programme improved body mass index (BMI), plasma insulin and triglyceride levels in both sexes, while the effect on microRNA expression profile was dissimilar between men and women. In females, at the end of the KD programme, miR-148b-3p, miR-26a-5p, miR-30e-5p, miR-502-5p, miR-520 h, miR-548d-3p, miR-590-5p and miR-644a were significantly different compared to the baseline; while in males a change in expression was observed for miR-30e-5p, miR-502-5p, miR-590-5p and miR-644a. Interestingly three microRNAs, let-7b-5p, miR-143-3p, miR-504-5p, showed the same statistical difference between baseline and 6 weeks of KD in both sexes. Bioinformatics analysis revealed that the differentially expressed microRNA target genes were linked to cytokine signalling pathways, inflammation and immunity, nutrient metabolism, oxidative phosphorylation, PPARs functional regulation and insulin signalling pathways [76]. Margolis et al., instead focused on the modulation of selected skeletal-muscle specific microRNAs in serum (c-myomiR: miR-1, miR-133a-3p, miR-133b, miR-206) and their relationship with protein synthesis rates. In detail, 16 elderly men (mean age 64 ± 2 years) were first subjected to a first 7-day period of eucaloric weight maintenance (WM) diet, to allow adaptation to the subsequent 28-day 30% energy-restricted (ER) diet. In the ER period, the authors showed not only an up-regulation of the c-myomiR score, with significant increase of miR-133a-3p and miR-133b, but also an inverse correlation between this score and the whole body protein synthesis rate, suggesting that ER may have a negative impact on skeletal-muscle regeneration, the top predicted target process at bioinformatics analysis. However, this study has several limitations, including the small number of participants, the lack of muscle biopsy analysis and body composition evaluation, such as fat-free mass [77].

Parr et al., evaluated plasma expression of 13 selected microRNAs, previously shown to be modulated by energy restriction and with putative roles in weight loss, at baseline and after a 16-week diet and physical activity intervention. The enrolled patients (*n* = 111) were subjected to physical exercise and randomly assigned to three dietary groups with different macronutrient intake: high dairy protein and carbohydrate (HPHC), high dairy protein and moderate carbohydrate (HPMC), low dairy protein and high carbohydrate (CON). Circulating microRNAs were analysed in 40 patients and further divided into two subgroups on the basis of body mass reduction: “high responders”, HiRes, ≥ 10% (*n* = 8 HPMC, *n* = 5 HPHC, *n* = 9 CON) and “low responders”, LoRes, ≤ 5% (*n* = 6 HPMC, *n* = 9 HPHC, *n* = 3 CON). In accordance with the above findings reported by Milagro et al., higher levels of miR-935 were observed in LoRes compared to HiRes at baseline and miR-140, miR-221 and miR-223 expression increased after 16-week weight-loss intervention, suggesting a putative role of these microRNAs as biomarkers of variability in individual response to weight-loss interventions. Interestingly, miR-935 was the only microRNA already higher at baseline in the LoRes group whose significant difference was maintained until the end of the intervention period. However, an important limitation of this study is related to the combination of dietary regimens and physical exercise that does not allow to evaluate the impact of the single intervention on the selected circulating microRNA profile [78]. The effects of exercise combined with dietary intervention has indeed been evaluated in other studies. One of them focused on the modulation of endothelial function in a population of obese adolescents. This study was carried out on an experimental group (EG) of 37 obese male adolescents (12–18 years) and a control group (CG, *n* = 10) of normal weight adolescents. The intervention was based on a 6-week calorie restriction diet associated with aerobic exercise. Regression analysis indicated a positive correlation between changes in serum miR-126 levels and BMI, serum levels of endothelium-derived nitric oxide (NO) and endothelin-1 (ET-1), before and after dietary intervention in the EG respect to CG. These data suggest that miR-126 might be involved in the improvement of microvascular endothelial function measured by RHI index [79].

### microRNA expression in adipose tissue

**Fig. 1:**
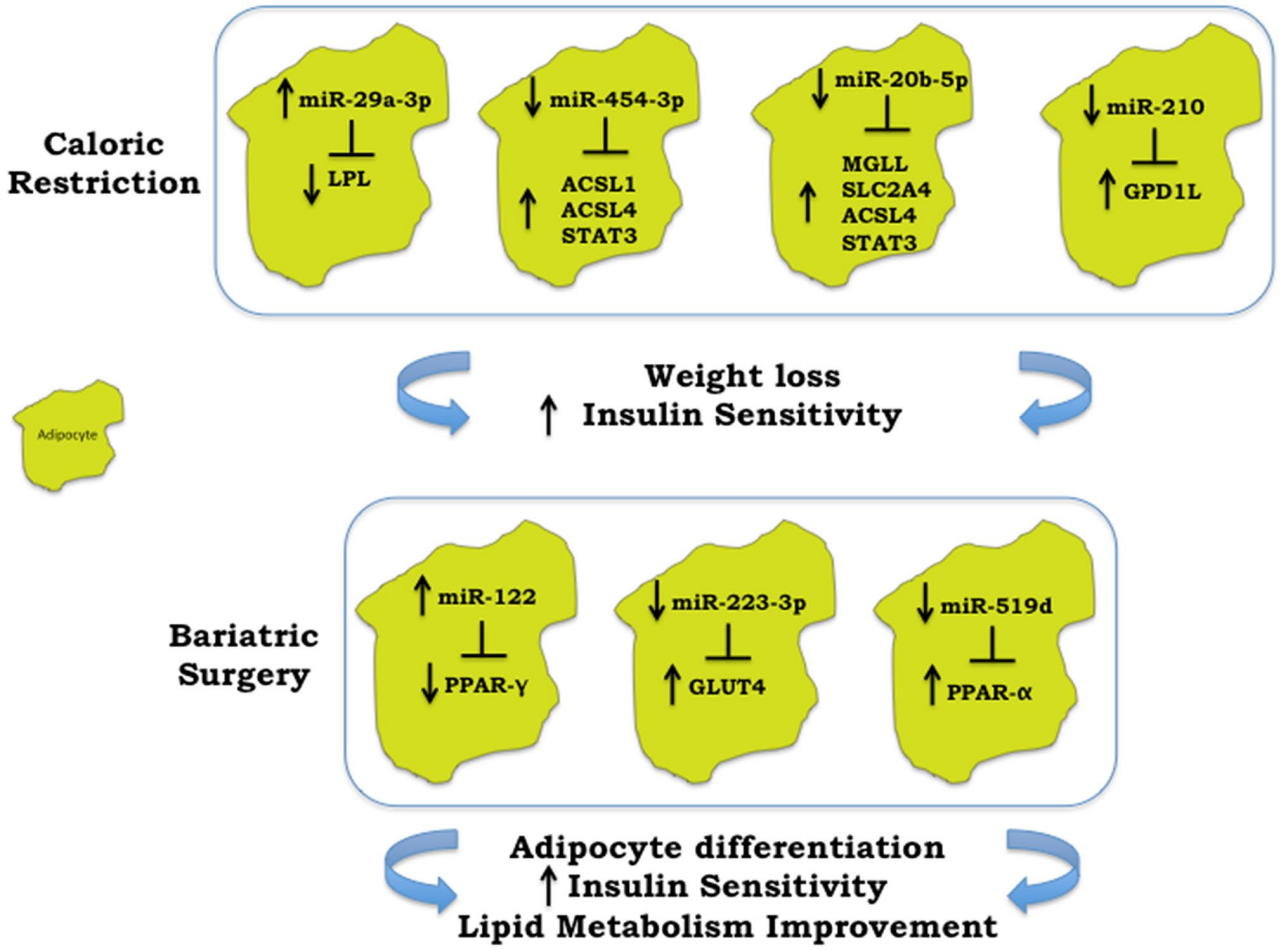
Caloric restriction and/or bariatric surgery modulation of adipose tissue microRNAs. **a** Caloric Restriction. The increase of miR-29a-3p after caloric restriction was paralleled with lipoprotein lipase (LPL) reduction, while the reduction of miR-454-3p, miR-20b-5p and miR-210 were paralleled with the increase of other pivotal players in controlling weight loss and insulin resistance. Specifically, acyl-CoA synthetase long-chain family member 1 (ACSL1) up-regulation was related with miR-454-3p down-regulation. Monoglyceride lipase (MGLL) and solute carrier family 2 member 4 (SLC2A4) were related with miR-20b-5p modulation. While acyl-CoA synthetase long-chain family member 4 (ACSL4) and signal transducer and activator of transcription 3 (STAT3) up-regulation was related with both miR-454-3p and miR-20b-5p down-regulation. The reduction of miR-210 was coupled with Glycerol-3-Phosphate Dehydrogenase 1 Like (GPD1L) increase. **b** Bariatric surgery. The increase of miR-122 was linked with peroxisome proliferator-activated receptor γ (PPAR-γ), while miR-223-3p and miR-519d were related with glucose transporter 4 (GLUT-4) and PPAR-α up-regulation, respectively

Kristensen et al., analysed microRNA expression in SAT derived from 19 obese patients undergoing a 15-week weight-loss strategy including both physical exercise and a hypocaloric dietary regimen. They identified nine differentially expressed microRNAs after intervention, but the statistically significant modulation of only three of these microRNAs was confirmed by validation on a wider cohort of patients (*n* = 38). In detail, miR-29a-3p and miR-29a-5p were up-regulated, while miR-20b-5p and miR-454-3p were down-regulated (the latter only had a borderline down-regulation), comparing the baseline with the post-intervention expression. In addition, the authors identified 56 predicted target genes of these four validated microRNAs and noticed a statistically significant inverse correlation between acyl-CoA synthetase long-chain family member 1 (ACSL1) with miR-454-3p; monoglyceride lipase (MGLL) and solute carrier family 2 member 4 (SLC2A4), which is the main responsible for glucose uptake into adipocytes with miR-20b-5p; lipoprotein lipase (LPL) with miR-29a-3p and finally acyl-CoA synthetase long-chain family member 4 (ACSL4) and signal transducer and activator of transcription 3 (STAT3) with both miR-454-3p and miR-20b-5p (Fig. 1).

This last observation is interesting since these two microRNAs may act together in the modulation of genes, such as ACSL4 and STAT3, involved in energy homeostasis and metabolism [80]. Other studies that analysed microRNA expression in SAT have been conducted. In detail, He et al., examined possible molecular mechanisms underlying obesity through the integration of genetic obesity-associated loci derived from the largest genome-wide association study (GWAS) meta-analysis for BMI, gene expression and microRNA profiles, in adipose tissue from 200 subjects. This approach was further supported by the analysis of five publicly available studies comparing obese and non-obese subjects who underwent different dietary intervention strategies. It is worth noting that He et al., identified a molecular axis involving hypoxia/Glycerol-3-Phosphate Dehydrogenase 1 Like (GPD1L)/miR-210 that can be linked to fat accumulation and obesity. GPD1L has been recently demonstrated to be a regulator of HIF-1α stability and a validated target of miR-210, a master regulator of hypoxia. Since adipose tissue in patients is scarcely oxygenated, with a consequent relative hypoxic status, the authors hypothesized that the increase of HIF-1α expression and activity in high fat diet (HFD) models could induce an up-regulation of miR-210, with the subsequent reduction in GPD1L levels and a further repression of prolyl hydroxylases (PHDs) activity. All these events may determine the activation of a feedback loop that further promote HIF-1α accumulation, with subsequent worsening of obesity, insulin resistance and adipose tissue inflammation. Interestingly, low calorie diet was able to induce a relative reduction in the hypoxic state and of miR-210 levels, paralleled with an increase of GPD1L levels (Fig. 1) [81–83]. Giardina et al. [71] instead evaluated the microRNA profile in a randomly selected representative cohort of eight patients subjected to different dietary interventions, previously described. The 13 most differentially expressed microRNAs in SAT were further selected for validation in a wider cohort of 48 patients. The expression of miR-551b, miR-221, miR-378 and let7a was down-regulated after the LGI intervention. The expression of miR-1276, miR-132 and miR-29a was down-regulated after the HGI intervention, while the expression of miR-661, miR-1179, miR-132, miR-221, miR-29a and miR-378 was down-regulated after the LF intervention. Each dietary approach induced a significant reduction in body weight, BMI, waist circumference, fat mass, fat-free mass and HOMA-IR compared to the respective baseline values. Moreover, the expression of some microRNAs, namely miR-551b, miR-1179, miR-132, miR-221, miR-29a, miR-34a, miR-378, correlated positively with biochemical and anthropometric variables, indicating that weight loss and changes in body composition, rather than dietary composition, could be the main drivers in the modulation of microRNAs profile [84].

## microRNAs and weight loss: evidence from bariatric surgery intervention studies

A wide number of studies focused on the possible relationship between microRNA expression and bariatric surgery (Table 2), highlighting that surgically induced weight loss is effective in rescuing microRNA signature [85].

**Table 2:** Studies that reported changes in microRNA expression after bariatric surgery

Study	Population	Intervention	Source	Regulated miRNAs	Role/target
Hulsmans et al. 2012 [86]	9 obese 6 CTRL	RYGB	PBMC	miR-181 (↑)	TLR-NFkB pathway
Ortega et al. 2013 [70]	6 obese	RYGB	Plasma	miR-16-1, miR-122, miR-140-5p, miR-193a-5p (↓) miR-221 and miR-199a-3p (↑)	–
Nunez-Lopez et al. 2017 [87]	22 obese	RYGB	Plasma	miR-15a (↑) miR-34a, miR-122 (↓)	Biomarkers of weight loss /glucose metabolism
Atkin et al. 2018 [88]	29 T2D	RYGB	Plasma	miR-7-5p, let-7f-5p, miR-15b-5p, miR-320c, miR-205-5p, miR-335-5p (↑) let-7i-5p (↓)	Inflammation, adipocyte proliferation, ß-cell function, thyroid and pituitary function
Hubal et al. 2017 [92]	6 obese women	RYGB	Plasma and serum adipocyte-derived exosomes	let-7a-5p, miR-16-5p	Insulin signalling
Bae et al. 2019 [93]	16 obese 18 CTRL	LSG (*n* = 2) RYGB (*n* = 14)	Serum exosomes	miR-424-5p	Biomarker of weight loss
Macartney-Coxson et al. 2020 [94]	15 obese women	RYGB	SAT VAT	SAT: miR-23a-5p, miR-27a-5p, miR-200c-3p, miR-223-3p, miR-1246, miR-24-2-5p, miR-128, miR-421, miR-3178, miR-1224-5p, miR-221, miR-22, miR-762 (↓) VAT: miR-223-3p (↓)	Inflammation, glucose uptake
Liao et al. 2018 [98]	20 obese 8 CTRL	LSG	SAT VAT	VAT: miR-122 (↑)	PPAR-γ
Kurylowicz et al. 2016 [99]	20 obese 7 CTRL	Bariatric surgery	SAT	miR-146b-3p, miR-146b-5p, miR-223-3p, miR-223-5p, miR-941 (↑)	BMPR2, FOXP1, IGF1R
Ortega et al. 2015 [100]	16 obese	RYGB	SAT	miR-155, miR-221, miR-130b (↓)	Inflammation
Ortega et al. 2015 [101]	9 obese women	RYGB	SAT	miR-19a/b, miR-146a/b, miR-155, miR-193b, miR-221, miR-222, miR-223, miR-376c, miR-411 (↓)	Glucose uptake, lipid metabolism, energy homeostasis
Nardelli et al. 2017 [102]	3 obese 2 CTRL	LAGB	SAT	miR-519d, miR-299-5p, miR-212, miR-671-3p (↓) miR-370, miR-487a (↑)	PPAR-α (miR-519d)

CTRL, control subjects; RYGP, Roux-en-Y gastric by-pass; PBMC, peripheral blood mononuclear cells; T2D, type 2 diabetes; SAT, subcutaneous adipose tissue; VAT, visceral adipose tissue; LSG, laparoscopic sleeve gastrectomy; BMPR2, bone morphogenic protein receptor 2; FOXP, forkhead box protein P1; IGF1R, insulin-like growth factor receptor 1; LAGB, laparoscopic adjustable gastric banding

### microRNA expression in white blood cells

Changes in inflammatory toll-like receptor (TLR)/nuclear factor kB (NFkB) related microRNAs have been evaluated in circulating monocytes from obese subjects undergoing RYGB and lean controls. Microarray analysis identified 133 differentially expressed microRNAs in circulating monocytes of obese subjects (*n* = 9) compared with lean controls (*n* = 6). In addition, target prediction revealed that nine dysregulated microRNA families were associated with TLR/NFkB pathway, among them, in a larger validation cohort of 14 lean controls and 21 morbidly obese patients, only the miR-181 family resulted to be down-regulated in monocytes of obese patients and its dysregulated levels were restored after bariatric surgery [86].

### Circulating microRNAs

In the context of bariatric surgery, several studies focused on the expression pattern of circulating microRNAs before and after surgical intervention. In 2013, Ortega et al., analysed the differences in the microRNA expression profile before and after RYGB in plasma samples from six morbidly obese patients. The results were validated in an independent cohort of 22 obese patients and 14 circulating microRNAs resulted significantly modulated. In particular, the authors observed a marked reduction in miR-16-1, miR-122, miR-140-5p, miR-193a-5p and an up-regulation of miR-221 and miR-199a-3p [70].

Nunez-Lopez et al., were interested in the identification of non-invasive biomarkers for metabolic changes resulting from RYGB. They recruited 22 morbidly obese subjects who had undergone RYGB surgery 1–3 months before. At baseline evaluation, subjects were divided in two subgroups: 11 subjects were subjected to a 6-month exercise-programme (EX) and the other 11 to a control health education intervention (CON). Interestingly, 94 plasma microRNAs, selected on the basis of their relationship with metabolism, were analysed. The authors identified three patterns of circulating microRNA changes in the patient cohort at the end of the intervention. Specifically, three microRNAs were modulated both in the CON and in the EX group (miR-15a increased, while miR-34a and miR-122 decreased), suggesting that their modulation may indicate a general response to weight loss induced by RYGB surgery; three microRNAs were modulated only in the CON group (miR-7 and miR-106 increased, while miR-221 decreased) and four microRNAs only in the EX group (miR-135b, miR-144 and miR-206 decreased, while miR-149 increased). Interestingly, the 10 above mentioned microRNAs significantly correlated with indices of HOMA-IR, β-cell function, body composition, plasma lipids and liver function. Moreover, miR-15a, miR-7, miR-106b and miR-135b correlated, already at the baseline, with insulin sensitivity (miR-15a), glucose effectiveness (miR-7 and miR-106b), and acute insulin response to glucose (miR-135b), suggesting their role as predictive biomarkers of cardiometabolic changes [87].

Atkin et al., analysed plasma microRNA expression in obese patients (*n* = 29) with T2D, undergoing RYGB. Six microRNAs (miR-7-5p, let-7f-5p, miR-15b-5p, miR-320c, miR-205-5p, miR-335-5p) significantly increased, while let-7i-5p decreased after surgery. Interestingly, a marked reduction in blood glucose and glycated haemoglobin (HbA1c) levels was observed after surgery in a high percentage of patients (more than 60%) [88]. Most of the regulated microRNAs were found to be expressed in natural killer cells, which play critical roles in obesity-induced inflammation [89]. Furthermore, the same microRNAs have been shown to be involved in different biological processes, such as adipocyte proliferation, ß-cell function, thyroid and pituitary function [90, 91].

Hubal et al., explored plasma and serum adipocyte-derived-exosomal microRNAs in six obese women before (T0) and 1-year (T1) after RYGB. Interestingly, 29 differentially expressed microRNAs between T0 and T1 were significantly correlated to improvement in the HOMA-IR. Pathway analysis identified the Insulin Receptor Signalling as one of the most enriched pathways. Moreover, two of the above mentioned microRNAs (let-7a-5p, miR-16-5p) correlated not only to the Insulin Receptor Signalling, showing the highest number of targets in this pathway, but also to branched chain amino acids (BCAAs) levels, which are strictly connected to insulin dysregulation. These findings indicate that changes in circulating adipocyte-derived exosomal microRNAs may be connected with post-surgery improvements in glucose homeostasis and insulin resistance [92].

Other authors explored circulating microRNAs in serum exosomes obtained from obese patients (*n* = 16), before and 6 months after bariatric surgery, and from healthy subjects (*n* = 18). Nine exosomal and 32 circulating microRNAs displayed higher and lower expression, respectively, in obese patients after intervention compared to baseline. Remarkably, among the nine up-regulated microRNAs, the levels of miR-424-5p before surgery positively correlated with weight loss after intervention [93]. Due to their high stability, exosomal microRNAs are novel candidate biomarkers for several pathological conditions. Since a considerable number of exosomal microRNAs decreased after bariatric surgery, in parallel with the reduction in fat mass, it can be speculated that most circulating exosomal microRNAs derive from adipose tissue.

### microRNA expression in adipose tissue

Although mounting evidence has focused on circulating microRNA profiling to individuate candidate biomarkers of response to weight loss after dietary intervention or bariatric surgery, a debate is still on-going on whether circulating microRNA dysregulation directly reflects changes at a tissue or cellular level. In light of this, several studies focused on the differences in microRNA expression profile between SAT and VAT, before and after bariatric surgery.

Recently, microRNA expression has been explored in SAT and VAT of obese women (*n* = 15) collected both during RYGB and then after 17 months, at the time of a second surgery for other purposes. Several microRNAs, namely miR-23a-5p, miR-27a-5p, miR-200c-3p, miR-223-3p, miR-1246, miR-24-2-5p, miR-128, miR-421, miR-3178, miR-1224-5p, miR-221, miR-22 and miR-762, were significantly down-regulated after surgery in SAT, while only miR-223-3p showed significant down-regulation in VAT [94]. Selected targets of miR-223-3p, which have been found to be over-expressed in obesity [95], have been evaluated. In both tissues, NLRP3 (NACHT, LRRand PYD domains-containing protein 3) and leptin (LEP) mRNAs were significantly down-regulated, while glucose transporter 4 (GLUT-4) was up-regulated after surgery. Furthermore, the GLUT-4 mRNA positively correlated to miR-223-3p expression in SAT (Fig. 1) [94]. Remarkably, the NLRP3 consists of a set of intracellular sensors and receptors, known as inflammasome, recognized to act as key contributors to the chronic inflammatory state observed in obesity [96].

Liao et al., compared the microRNA expression pattern in SAT and VAT in 20 patients undergoing LSG and 8 normal weight subjects undergoing laparoscopic cholecystectomy. SAT and VAT from obese patients were characterized by 18 differentially expressed microRNAs: 12 were significantly up-regulated and 6 down-regulated in VAT. Conversely, these differences were less marked in non-obese subjects. Among the differentially expressed microRNAs, miR-122 was the most up-regulated in VAT from both obese and non-obese subjects in respect to SAT, and its over-expression was confirmed by RT-qPCR in a wider cohort of patients. By over-expressing this microRNA in fully differentiated mouse 3T3-L1 cells, the authors identified the peroxisome proliferator-activated receptor γ (PPAR-γ) as the most significantly altered pathway. Moreover, the over-expression of miR-122 induced a significant reduction in PPAR-γ mRNA, while its inhibition led to a significant increase in PPAR-γ mRNA expression levels, suggesting that miR-122 regulates PPAR-γ, one of the most important players in adipocyte differentiation (Fig. 1) [97, 98].

Kurylowicz et al., instead compared microRNA profile before and after bariatric surgery only in SAT. In detail, they analysed the microRNA profile in VAT and SAT from 10 obese patients (O), VAT and SAT from 7 normal weight subjects (N) and SAT from 10 obese patients about 2-years after surgery-induced weight loss (PO). By focusing on the differences in microRNA expression between SAT samples before and after weight loss (SAT-O vs SAT-PO), the authors identified 58 over and 3 under-expressed microRNAs in SAT-O compared with SAT-PO. Five of them, namely miR-146b-3p, miR-146b-5p, miR-223-3p, miR-223-5p and miR-941, were also under-expressed in SAT-N in respect to SAT-O. However, there were also differences in the microRNA expression pattern between SAT-PO and SAT-N samples. In particular, 79 microRNAs were differentially expressed: 42 were under-expressed, whereas 37 over-expressed in SAT-PO in respect to SAT-N, indicating that obesity may determine persistent miRNome changes. Finally, the authors reported that several targets with well-known roles in obesity, such as the bone morphogenic protein receptor 2 (BMPR2), the forkhead box protein P1 (FOXP1) and the insulin-like growth factor receptor 1 (IGF1R), comprised the highest number of binding sites in the differentially expressed microRNAs [99].

The SAT microRNA profile of obese subjects was also analysed by other authors. In 2015, Ortega et al. evaluated the microRNA profile changes in SAT from obese subjects after laparoscopic RYGB. The first study enrolled 16 obese patients in which 15 microRNAs showed significant modification after surgery-induced weight loss. Among them, miR-155, miR-221 and miR-130b displayed decreased expression after treatment [100]. The same authors identified significant reduction in 11 microRNAs (miR-19a/b, miR-146a/b, miR-155, miR-193b, miR-221, miR-222, miR-223, miR-376c, miR-411) in SAT samples of nine morbidly obese women after surgery-induced weight loss. Interestingly, three of them, namely miR-155, miR-221 and miR-222, were associated with obesity-related inflammation and weight loss, suggesting that microRNA modulation after weight loss may underline improvement in patients’ inflammatory status [101]. Subsequently, in 2017 Nardelli et al., analysed microRNA expression pattern in SAT obtained from three severely obese women before (T0) and three years after LAGB (T1), and from two lean subjects, undergoing laparoscopic cholecystectomy. Specifically, at T0, 43 microRNAs resulted significantly up-regulated and 15 down-regulated between obese and lean subjects. Of the above cited microRNAs, four resulted down-regulated (miR-519d, miR-299-5p, miR-212, miR-671-3p) and two up-regulated (miR-370, miR-487a) also in T1 compared to T0. MiR-370, miR-487a and miR-519d dysregulation was also confirmed by validation assays. Interestingly, the level of PPAR-α protein, a validated target of miR-519d [53], increased at T1 compared to T0, suggesting that miR-519d targets PPAR-α in SAT and may be involved in the improvement of lipid metabolism and SAT function after surgery (Fig. 1) [102].

Overall, in a meta-analysis of 17 studies evaluating the differential expression of microRNA before and after bariatric surgery both in humans and in animal models, several microRNAs were reported to be consistently down-regulated (miR-93-5p, miR-106b-5p, let-7b-5p, let-7i-5p, miR-16-5p, miR-19b-3p, miR-92a-3p, miR-222-3p, miR-142-3p, miR-140-5p, miR-155-5p, miR-320-3p) or up-regulated (miR-7-5p and miR-320c), with high concordance between studies [103]. However, it is worth highlighting that marked differences between the selected studies have emerged (i.e. population, type of intervention, follow-up length, collected samples) and further research is therefore needed to draw firm conclusions.

## Conclusions and future perspectives

Over the last 20 years, obesity and obesity-related disorders have rapidly become a public health concern worldwide. Recently, microRNAs have gained much attention as epigenetic modulators in obesity, helping understand the pathophysiological mechanisms underlying this condition. Several microRNAs have been found to be differentially regulated in obesity and their expression was significantly modified by different weight-loss approaches, such as diet, physical activity and bariatric surgery. Specifically, the above mentioned studies explored microRNAs expression in several compartments, such as blood cells, serum, plasma, adipose tissue, and the effect of a wide variety of currently available intervention strategies for obesity treatment has been evaluated. In particular, dietary interventions with marked differences in macronutrient composition have been compared, highlighting different effects on microRNA expression profile. These findings suggest that weight loss obtained through entirely different dietary strategies might be mirrored by profound changes in microRNA signature. As regards bariatric surgery, it should be highlighted that the vast majority of the available intervention studies have focused on the effect of the malabsorptive procedure RYGB, whereas data on other widely adopted surgical approaches, such as LSG and LAGB, are still limited. Future research is therefore needed to address this issue to possibly compare the effect of different surgical interventions on circulating and tissue microRNAs expression. Of note, the assessment of microRNA profile in adipose tissue before and after obesity treatment approaches gave critical information about the metabolic changes occurring in response to weight loss. Indeed, the definition of the mechanisms through which microRNAs regulate adipose tissue metabolism is of utmost interest, as it can lead to significant improvement in obesity treatment. Importantly, diverse patterns of microRNA expression between VAT and SAT have been observed in several studies, suggesting that weight loss might differently modify the metabolic profile of these tissues.

Furthermore, substantial differences in microRNAs expression have emerged between responders and non-responders to dietary and surgical interventions. Considering the consistent variability in individual response to weight-loss interventions, circulating microRNAs might be valuable biomarkers of efficacy, possibly helping in the differentiation between responders and non-responders. Remarkably, a wide variety of dysregulated microRNA target pathways, some of them crucially involved in glucose and lipid metabolism, energy homeostasis, inflammation, immunity, endothelial function, have also been identified, helping understand the pathophysiological mechanisms underlying obesity and obesity-related metabolic diseases.

It should be highlighted that some discrepancies emerged between studies. Nevertheless, these conflicting results might be attributed to several factors, such as heterogeneity of the included populations, different samples collected (plasma, serum, adipose tissue), type of intervention adopted (diet composition, physical activity interference, surgical procedure), variability in the analytic methods for microRNA profiling. As regards the latter, in future studies a strong effort should be made in analysing microRNAs through absolute quantification and RNA identification. Indeed, applying standardized techniques for RNA extraction and performing highly multiplexed single molecule counting would be a crucial advance in analytical methodologies.
